# A Joyful Christmas

**Published:** 1918-12

**Authors:** 


### A Joyful Christmas.

There has never before been a time when the people of the United States have had so much cause for rejoicing as at the end of 1918, a year into which the world entered with gravest forebodings and fears as to what it might mean for all civilization. Few Americans entertained any doubts as to the final results, but many feared that we might be years in bringing Germany to her senses, and that the victory might cost the lives of hundreds of thousands of American soldiers.

Many have made the supreme sacrifice and thousands of hearts are bleeding because loved ones have gone, but the country is fortunate in not having ten times the number killed or wounded. We should rejoice that the boys, except those who died over there, are coming back and that more will not have to be sent over to fight for permanent world peace.

The strain of war, as war is now made, is something fearful. Soldiers and civilians alike are called upon to suffer and to sacrifice. The work of ages is destroyed in a night and war-stricken countries are laid low in a day. The destruction of life and property is too horrible to contemplate.

But our greatest cause for rejoicing is not that war is over sooner than was expected, but rather that it has ended in such a way as, with the exercise of cool judgment, leads us to believe that arrangements will be perfected that there may be no more wars. Fortunate indeed is the world that the United States has at the head of its government a man with the brains and the heart to help to give to the whole world for all time that peace for which he has so long hoped.

Some of us may have lost loved ones, most of us have sacrificed until the strain of sacrifice has hurt, but it will not be in vain if the world can be made safe from future wars. Otherwise the struggle will be for naught.

